# Stability bounds on superluminal propagation in active structures

**DOI:** 10.1038/s41467-022-28713-x

**Published:** 2022-03-02

**Authors:** Robert Duggan, Hady Moussa, Younes Ra’di, Dimitrios L. Sounas, Andrea Alù

**Affiliations:** 1 Department of Electrical and Computer Engineering, The University of Texas at Austin, Austin, TX 78712 USA; 2 Photonics Initiative, CUNY Advanced Science Research Center, New York, NY 10031 USA; 3 Department of Electrical Engineering, City College of the City University of New York, New York, NY 10031 USA; 4 Department of Electrical and Computer Engineering, Wayne State University, Detroit, MI 48201 USA; 5 Physics Program, Graduate Center, City University of New York, New York, NY 10026 USA

**Keywords:** Transformation optics, Nanophotonics and plasmonics

## Abstract

Active materials have been explored in recent years to demonstrate superluminal group velocities over relatively broad bandwidths, implying a potential path towards bold claims such as information transport beyond the speed of light, as well as antennas and metamaterial cloaks operating over very broad bandwidths. However, causality requires that no portion of an impinging pulse can pass its precursor, implying a fundamental trade-off between bandwidth, velocity and propagation distance. Here, we clarify the general nature of superluminal propagation in active structures and derive a bound on these quantities fundamentally rooted into stability considerations. By applying filter theory, we show that this bound is generally applicable to causal structures of arbitrary complexity, as it applies to each zero-pole pair describing their response. As the system complexity grows, we find that only minor improvements in superluminal bandwidth can be practically achieved. Our results provide physical insights into the limitations of superluminal structures based on active media, implying severe constraints in several recently proposed applications.

## Abstract (web summary)

Wave packets can travel faster than the speed of light only under severe constraints on their bandwidth and propagation distance. The authors demonstrate stringent bounds for superluminal propagation, and their implications for various technologies.

## Introduction

Arguably one of the most fundamental laws of physics is that light in free space travels at speed ${c}_{0}$ in any inertial frame^1^. Given that the response of a material cannot precede the excitation field, causality requires the photon speed to remain below ${c}_{0}$ in any causal material. While of great interest from both fundamental and applied viewpoints, faster-than-${c}_{0}$ and even negative group velocities have repeatedly been shown to comply with severe constraints associated with relativity and causality, most notably by Sommerfeld^2^ and Brillouin^3^, in particular associated with anomalous dispersion features. In passive structures, these responses are therefore necessarily narrowband and associated with large absorption, consistent with Kramers-Kronig relations^4,5^. The group velocity $\partial \omega /\partial k$ measures the speed at which the peak of a narrowband pulse centered at frequency $\omega$ travels in a medium with wave number $k$, which can indeed move through a finite distance at any velocity, faster than ${c}_{0}$ or even negative (implying that the center of mass of the pulse exits the system before entering it, due to distortion). The required presence of frequency dispersion and absorption guarantees however that exotic values of group velocity are always associated with large pulse distortions, with the pulse center shifting towards the precursor to avoid noncausal responses. The integrated energy exiting a system with anomalous group velocity at any instant in time is always lower than the one passing through free space^6^.

In the quest to suppress absorption and distortions in superluminal media, there has been a recent interest in using structures with gain. Breaking the assumption of passivity, it is indeed possible to envision frequency dispersion profiles that, while complying with Kramers-Kronig relations, offer superluminal or negative group velocities over large bandwidths. Optical gain^7–9^ and non-Foster circuit elements^10,11^ have been explored to enable inverted dispersion and define broadband superluminal propagation, opening opportunities in a number of practical scenarios, including enhanced bandwidth for small antennas^12^, broadband leaky-wave antennas that do not scan the angle with frequency^11^, and broadband electromagnetic cloaks^13^ defying to some extent the limitations imposed by passivity^14^.

At microwave frequencies, for which amplifiers and broadband gain are widely accessible, scientists have been able to observe superluminal velocities with minimal dispersion over broad bandwidths^10,15^. The fundamental challenge in these experiments, and in related theoretical works, is that active systems are inherently prone to instabilities, and defining the group velocity based on the propagation of a signal through a finite, typically short, distance does not necessarily ensure that the system remains stable when considering arbitrary excitation schemes, propagation over longer distances, or different loading conditions. In Ref. ^16^, the authors derived the causality constraints of a single amplifier, and then assumed that an array of such elements would remain necessarily causal. Similarly, in Ref. ^17^, the authors introduced a gain material obeying causality to obtain low-dispersion superluminal group velocity tailored for broadband cloaking, claiming that this technique can be applied to cloak an arbitrarily large object to a broadband signal. These assumptions are inherently misguided, as we discuss in the following, and it is imperative to consider not only the causality of the constitutive materials, but also the stability of the overall finite structure under analysis. A structure made of causal media may not be stable if it comprises a collection of active elements. For instance, adding a number of active lumped elements, independently stable^18^, or lengthening the slab of a gain medium^19^, is known to lead to instabilities. In frequency domain, instabilities are manifested by the emergence of transfer-function poles in the upper half of the complex frequency plane under an ${e}^{-i\omega t}$ time convention, yielding self-oscillations and unbounded outputs until saturation and nonlinear effects kick in. Such instabilities are facilitated in the case of reflecting discontinuities, which can add positive feedback to the system, highlighting the necessity of considering also the load in these discussions.

In this work, we derive bounds dictated by stability in the quest to realize superluminal group velocities in active media, inspecting the complex frequency response of these structures, and we describe their implications on the functionality of broadband devices based on these principles. Our work clarifies the general nature of superluminal propagation in active systems, and outlines fundamental limitations and important challenges in practical implementations for the benefit of various device functionalities, at the same time straightening recent unwarranted claims in the context of active systems that may be able to support broadband superluminal propagation.

## Results

### Causality limits

**Fig. 1:**
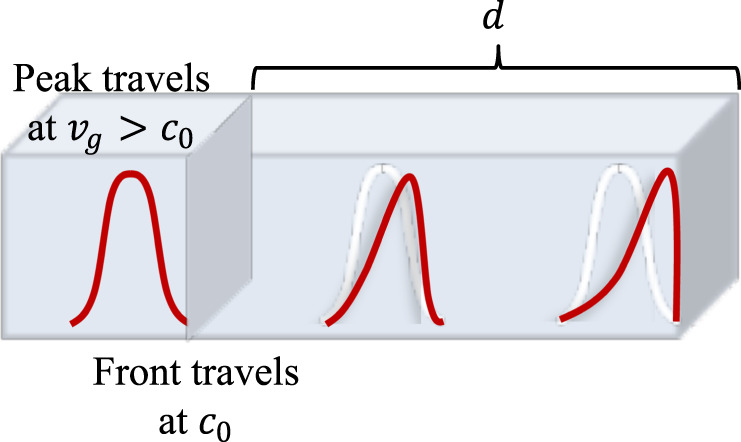
*Pulse propagation through a dispersive medium*. By causality, no portion of the impinging pulse, in particular its peak, which nominally travels at the group velocity, can surpass the forefront of the pulse. Since the temporal duration of the pulse is inversely proportional to its bandwidth, a pulse of narrower bandwidth should be able to support a larger superluminal velocity before the peak reaches the front. This example suggests a dependence of the allowed velocity on the bandwidth over which it can be achieved, and the propagation length before instabilities or distortions arise.

We start with a simple thought experiment to predict what sort of relationship exists between propagation velocity, length, and bandwidth that a slab of superluminal material can support. Consider a symmetrically-shaped pulse of duration $\varDelta t$ traveling in an unbounded medium for a length $d$, as in Fig. 1. Causality requires that the Sommerfeld forerunner^2^ reaches the end of the slab at ${t}_{0}={d/c}_{0}$, and no portion of the signal can arrive before then, hence no part of the pulse may pass the front. Applying this condition to the pulse peak, which passes the starting plane at $t=\varDelta t/2$ and travels at the group velocity, the system must satisfy the inequality

1 $$\frac{1}{{v}_{g}}\ge \frac{1}{{c}_{0}}-\frac{\varDelta t}{2d}$$

If the spatial extent of the input pulse is shorter than $d$, so that the entire pulse enters the slab before any feature leaves, a bound for the maximum velocity is

2 $$|{v}_{g}|\le \left|\frac{1}{\frac{1}{{c}_{0}}-\frac{\alpha }{d\,BW}}\right|,$$

where $BW=\frac{2\alpha }{\varDelta t}$ represents the pulse bandwidth, and $\alpha$ is a dimensionless proportionality coefficient. Equation (2) can also be written as $d\,BW\le \alpha {(1/{c}_{0}-1/{v}_{g})}^{-1}$, which shows that, because of causality, the maximum bandwidth over which the pulse peak can move at superluminal velocity is limited by the velocity and distance traveled inside the medium. As the signal bandwidth grows beyond the limits dictated by (2), the material dispersion kicks in and distorts the pulse shape to a point for which the same definition of group velocity loses its meaning and/or instabilities may arise.

The bound (2) poses a clear limit to the velocity that a medium can support before large distortions or instabilities kick in, and it must apply to any type of finite excitation in time. This is in contrast with claims that active superluminal structures are physical as long as causal materials are employed. More specifically, it has been argued that causal active media may support dispersionless responses with arbitrary superluminal velocity, bandwidth, and length, ideally suited for a variety of applications, e.g., for broadband cloaking devices^17^. The bound in Eq. (2), however, implies stringent constraints on the propagation velocity that a medium can sustain for arbitrary pulse excitation before distortions and nonlinearities necessarily kick in. In agreement with this expectation, active structures become unstable as their length is increased^18^. In Ref. ^15^, pulse reshaping arguments have been used to derive usable bandwidths over which superluminal propagation can be achieved, as a function of the acceptable level of pulse distortion. However, distortion usually depends on the input pulse, thus not permitting the derivation of bounds that exclusively depend on material properties. In the following, we derive a general bound stemming from stability considerations to determine a fundamental trade-off between velocity, bandwidth and propagation length in these structures, quantifying what can and cannot be done in the context of active media supporting superluminal propagation.

**Fig. 2:**
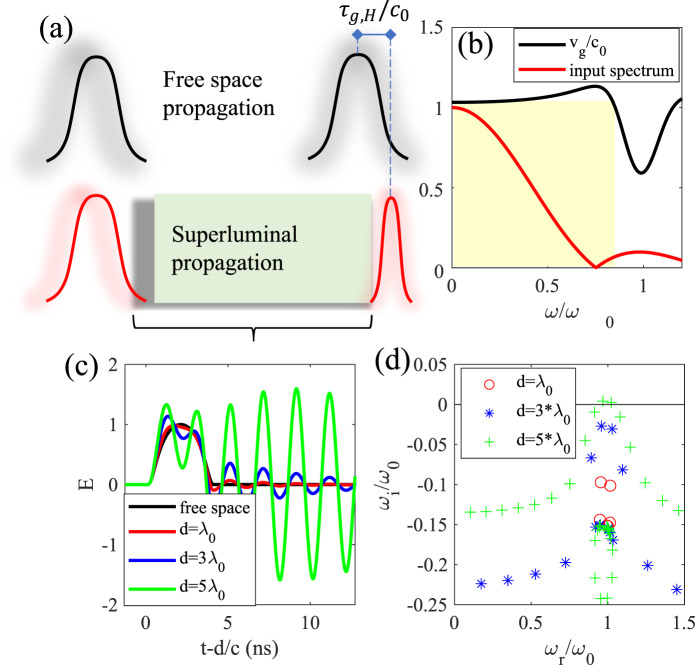
*Stability concerns in active superluminal strctures*. **a** Comparison of free-space propagation of a pulse (black) and propagation through a superluminal slab (red), where the peak of a pulse within the superluminal bandwidth advances by a time ${\tau }_{g,H}$ relative to free-space. **b** Group velocity (distance divided by group delay) of the considered active material and power spectrum of the input pulse. The highlighted box shows the bandwidth of superluminal propagation for which ${v}_{g}\ge {v}_{g}(\omega \to 0)$. **c** Time-domain signal transmitted through the slab, shifted by $d/{c}_{0}$. The signal is calculated by multiplying the input spectrum by the frequency-domain transfer function, and then inverse Fourier transforming into the time domain. The peak moves closer to the front of the pulse as the length increases, until we find an unstable response, unbounded for large times. **d** Poles of the transmission coefficient as a function of the slab length. The unstable response in (**c**) is associated with poles in the upper half-plane. Parameters: $A=-1/16$, $\gamma ={\omega }_{0}/3$.

As a starting point, we investigate the response of an active dielectric slab of finite thickness $d$ supporting a single inverted Lorentzian resonance, with relative permittivity ${\varepsilon }_{r}=1+\frac{A{{\omega }_{0}}^{2}}{{{\omega }_{0}}^{2}-{\omega }^{2}-i\gamma \omega }$, where $A \, < \, 0$ implies an active medium [Fig. 2(a)]. This model corresponds, for instance, to an inverted two-level system, and we assume no magnetic response (${\mu }_{r}=1$). The low-frequency group and phase velocities coincide, given by ${v}_{p,g}(\omega =0)=\frac{{c}_{0}}{\sqrt{1+A}}$, which is superluminal for active materials, providing a group velocity dispersion as in Fig. 2(b) (black line). Its Lorentzian dispersion ensures a causal impulse response, hence a wave traveling through an infinite sample of this material is expected to satisfy the constraints stemming from the relativity principle. This does not necessarily mean, however, that this material can be utilized in arbitrary configurations and be expected to maintain its linear causal features. To demonstrate the challenges that arise when a finite slab thickness of such material is considered, we numerically calculate the transmission^20^ of a smooth compact support pulse^21^ with spectrum [red line in Fig. 2(b)] mostly concentrated within the superluminal bandwidth of the structure [shaded in Fig. 2(b)], defined as the frequency range over which the length divided by the group delay through the structure is larger than ${c}_{0}$. Different from Fig. 1, here we consider the pulse impinging from free-space, for which mismatch and multiple reflections at the two interfaces become important. Independent of the slab thickness $d$, the pulse front is expected to exit the slab exactly at time $d/{c}_{0}$ because of causality (the Lorentzian dispersion ensures that the very high frequencies, corresponding to the pulse forefront, are indeed traveling at velocity ${c}_{0}$, as required by causality), but the pulse peak emerges closer and closer to the front, consistent with the superluminal group velocity. Correspondingly, the pulse is increasingly distorted, as shown in Fig. 2(c) for different slab thicknesses, including an amplifying oscillatory response [green curve, Fig. 2(c)] for sufficiently thick slabs. Once a critical length is reached (5*λ*_0_ in this example), consistent with Eq. (1), the poles of the transmission coefficient (i.e., the slab eigenfrequencies), plotted in Fig. 2(d), enter the upper half-plane, leading to an unstable response. The occurrence of this instability may be driven simply by noise, and so we can expect amplified oscillations for this slab even in the absence of a driven input, making the device unusable for any practical application.

Comparing this example with the previous calculations for an infinite medium that led to Eq. (1), the finite slab thickness adds reflections at the two interfaces, which can act as a positive feedback mechanism and make the active system prone to instabilities, despite the fact that the dispersion model of the bulk medium is causal. In a real system of finite spatial extent, we always expect interfaces and mismatched loads at least over some frequency ranges, so instabilities must always be carefully considered in analyzing active systems. In the unstable regime, the occurrence of nonlinearities and saturation effects eventually determines the actual overall response of the structure, but it is clear that a naïve analysis based on the response of the unbounded medium is bound to fail. Due to the scale invariance of Maxwell’s equations, we can also fix the slab thickness and instead vary the excitation and gain frequencies, yielding the same result as in Fig. 2, implying that claims of broadband superluminal propagation by relying on broadband material gain need to be carefully analyzed in the specific device implementation against the possible insurgence of instabilities.

Next, we explore fundamental limits on the superluminal bandwidth before instabilities set in. In passive systems, there are well-established global bandwidth limits for various functionalities of interest, such as the Bode-Fano matching bound^22,23^, the Rozanov absorption bound^24^, and similar bounds for cloaking^14^. These all stem from causality (the system poles must all be in the lower half plane), but also assume that, due to power conservation, the response is bounded (the reflection/transmission coefficient is always less than or equal to 1 for propagating waves). With active structures, the latter constraint does not hold, and so developing bounds becomes trickier. As we show below, there is no theoretical limit to the bandwidth over which superluminal propagation can be achieved in principle, but strict trade-offs and practical limitations arise in any realistic system.

### Filter theory limits

Filter theory^25^ offers general models to describe superluminal propagation in a variety of systems. For the sake of simplicity, we start by considering a one-dimensional 2-port network obeying causality, whose general transmission response can be written as

3 $$T(\omega )={e}^{\frac{i\omega d}{{c}_{0}}}\cdot H(\omega )$$

where $d$ is again the length of the structure, ${c}_{0}$ is the speed of light in free-space, and $H(\omega )$ is the structure-dependent response. The exponential term, corresponding to free-space propagation, is responsible for enforcing causality in the absence of any material response [$H(\omega )=1$] and the appropriate delay of inflection points, with frequencies approaching infinity expected to travel at ${c}_{0}$. The filter theory approach is usually performed in the Laplace *s*-plane^25^, but we utilize the complex $\omega ={\omega }_{r}+i{\omega }_{i}$ plane to be consistent with the previous formulation. Stability restricts the poles of $T(\omega )$, and therefore $H(\omega )$, to the lower half-plane (${\omega }_{i} \, < \, 0$), and the real nature of the fields [$T(\omega )={T}^{\ast }(-\omega )$] dictates that poles and zeros exist in pairs of opposite real part but same imaginary part, or otherwise lie on the imaginary axis (${\omega }_{r}=0$). To ensure a bounded $|T|$ at high frequencies, the number of zeros must not exceed the number of poles.

**Fig. 3:**
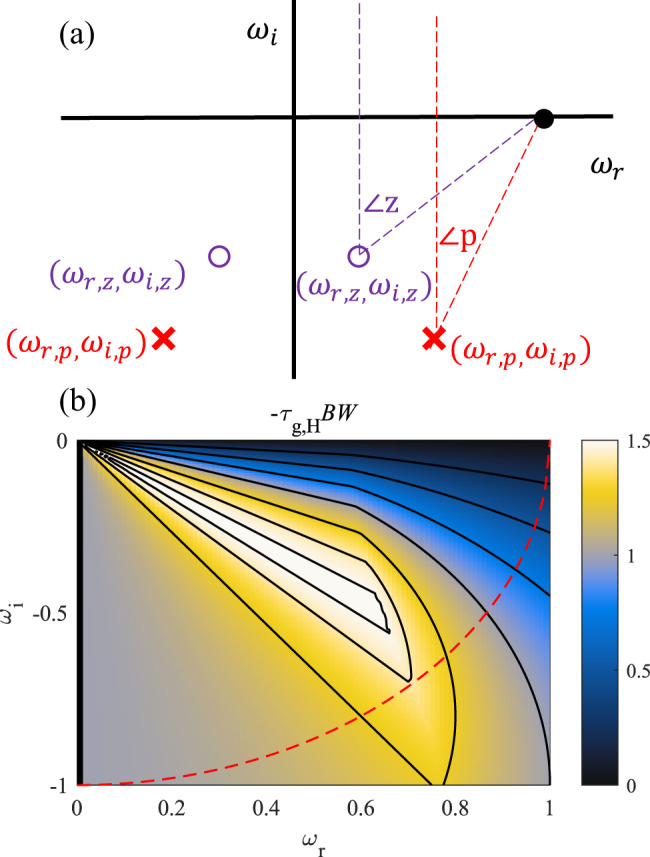
*Filter Theory Limits for a Single Pole-Zero Pair.* (**a**) Depiction of the angle definitions for our derivation of the filter-theory bound, where the circles indicate transfer function zeros and “x”s represent poles. **b** Minimum group-advance-bandwidth product as a function of position of the zero in the complex frequency plane. The pole defines the bandwidth, and it is located at $\omega =1$. The maximum advance-bandwidth product is found to be 3/2. For unitary transmission at $\omega =0$, the zero must lie on the red curve in the figure, along which the maximum value is $\sqrt{2}$. The corresponding pole-zero pairs also exist at mirror positions around the imaginary axis. The solid black lines indicate (unequal, for illustrative purposes) contours of $[0.25,0.5,0.75,1,1.25,\sqrt{2},1.49]$.

For any real frequency ${\omega }_{r}$, the transmission phase $\theta$ is given by

4 $$\theta =\frac{{\omega }_{r}d}{{c}_{0}}-\mathop{\sum}\limits_{zeros}\angle {z}_{i}({\omega }_{r})+\mathop{\sum}\limits_{poles}\angle {p}_{j}({\omega }_{r})={\theta }_{fs}+{\theta }_{H},$$

where the angles $\angle {z}_{i}({\omega }_{r})(\angle {p}_{i}({\omega }_{r}))$ are defined as the acute vertex formed by the $\omega$-plane points ${\omega }_{1}=i\infty ,\,{\omega }_{2}={z}_{i}({p}_{i}),\,{\omega }_{3}={\omega }_{r}$ [see Fig. 3(a)], and we split the phase into the free-space component and the one due to $H(\omega )$. The amplitude at a given frequency is the product of the distance from the zeros divided by the product of the distance from the poles, i.e., $|T({\omega }_{r})|=\mathop{\prod}\limits_{i}|{z}_{i}-{\omega }_{r}|/\mathop{\prod}\limits_{j}|{p}_{j}-{\omega }_{r}|$.

The group delay ${\tau }_{g}({\omega }_{r})=\frac{d\theta }{d{\omega }_{r}}={\tau }_{g,fs}+{\tau }_{g,H}$, written as the sum of the delays in free space and in the structure. The zeros contribute negatively to the group delay when in the lower half-plane, and the poles positively. In order to yield a broadband negative delay, the zeros should be optimally tailored to support a broadband response (with bandwidth increasing as the zero moves away from the real axis), but also have a fast angular derivative over the bandwidth of interest (implying they should not be too far from the axis). At the same time, to minimize the positive delays introduced by the poles, they should be positioned to have a small angular derivative over the operating bandwidth. This can either be achieved with poles with large negative ${\omega }_{i}$ values ($\angle {p}_{j}({\omega }_{r})\approx 0$), or placed near the ${\omega }_{r}$-axis outside the frequency range ($\angle {p}_{j}({\omega }_{r})\approx \pm \frac{\pi }{2}$). In the former case, the amplitude in the superluminal regime will be small, as in the case of anomalous dispersion in the presence of large losses. The latter requires active structures, as the system will exhibit gain at frequencies near the pole.

**Fig. 4:**
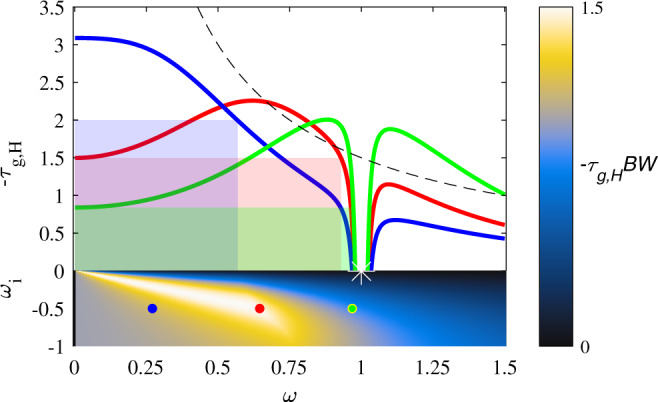
*Effect of pole position on group delay dispersion*. Dispersion curves for different positions of the zeros in the complex plane. In all plots, the pole is at the complex frequency point $(1,-0.001)$ (white star). The dotted line shows our bound, and the solid curves indicate the group advance for the pole positions given by the dots in the lower half-plane, where we have also included the same plot as Fig. 3(**b**). The shaded rectangles are the ones for the curve of the same color (blue, red, or greed) that maximize the $-{\tau }_{g,H}BW$ product.

We next define the figure of merit $-{\tau }_{g,H}BW$, which quantifies the normalized peak advance bandwidth product compared to propagation through an equivalent distance in free-space [as depicted in Fig. 2(a)]. With some algebra it can be found that this figure of merit is bounded by $\alpha$ based on Eq. (2). We define the bandwidth as the frequency range over which the smallest group advance is at least ${\tau }_{g,H}$. For one pair of complex-conjugate zeros, geometric optimization shows that this product cannot be larger than 3/2, a limit reached when the zero lies precisely on the line ${\omega }_{i}=-\sqrt{3/5}\,{\omega }_{r}$. Figure 3(b) shows the advance-bandwidth product $-{\tau }_{g,H}BW$ as a function of position of the zero in the complex frequency plane, after we assumed that the pole is placed on the real axis at ${\omega }_{r}=1$. In this case, the pole does not have an appreciable effect on the group delay for 0 < *ω*_*r*_ < 1. As the zero moves away from the origin on the line ${\omega }_{i}=-\sqrt{3/5}{\omega }_{r}$, the group advance at ${\omega }_{r}\to 0$ decreases, but the bandwidth correspondingly increases keeping the product constant. If the zero moves too far from the origin, the pole introduces a group delay within the bandwidth supported by the zero, reducing the total advance-bandwidth product. Figure 4 shows three examples of group-advance dispersion curves as the position of the zero is varied. We keep the pole at fixed position close to the axis, which has the effect of introducing a positive group delay (negative advance) for frequencies in its vicinity. The shaded regions indicate the corresponding $-{\tau }_{g,H}BW$. When the zero is too close to the imaginary axis, there is a large group advance, but over a sub-optimal bandwidth. If the real part of the frequency for the zero is too large, the velocity is smaller and the bandwidth is still limited by the pole’s position. At ${\omega }_{i}=-\sqrt{3/5}\,{\omega }_{r}$, corresponding to the red curve in Fig. 4, the maximum advance-bandwidth product is obtained.

This analysis assumes that the placement of pole and zero can be arbitrary, though practical considerations are expected to introduce additional limitations. For example, in a shunt impedance-inverted RLC circuit, as considered in Supplementary Discussion 1, the zero must lie on the unit circle due to its unitary gain at DC, corresponding to the red dashed curve in Fig. 3(b). Under this assumption, the maximum advance-bandwidth product is $\sqrt{2}$. To reach the limit $\alpha =3/2$, the zero must move off the unit circle closer to the origin, which means that we must introduce loss at $\omega =0$.

### Dielectric slab

**Fig. 5:**
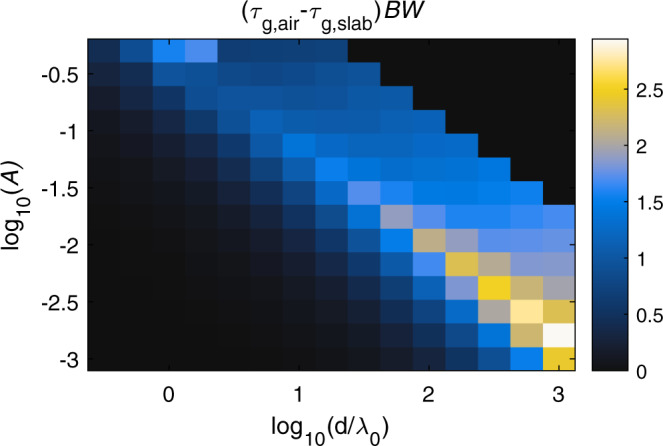
*Numerical limits*. Group delay-bandwidth product in an active superluminal dielectric slab relative to the delay in an equal thickness free-space slab. For given $d$ and $A$, $\gamma$ is varied to maximize the bandwidth, while ensuring that the structure remains inherently stable. For comparison, the bound for a single-pole RLC resonator is 1.5.

The derived limit applies to each zero of the transfer function of a linear system. Due to the linearity of (4), additional zeros in $H(\omega )$ can each in principle provide an additional 1.5 contribution to the total $-{\tau }_{g,H}BW$. With sufficient control over the system, the total advance-bandwidth product can therefore in principle be made large. Realistically though, a practical system cannot support arbitrary dispersion engineering, and limitations naturally arise. The dielectric slab considered earlier is a good example: its multiple Fabry-Pérot modes correspond to a large number of poles and zeros in the transmission coefficient, hence the limit of $\alpha =1.5$ does not have to hold, yet its value of $-{\tau }_{g,H}BW$ does not become much larger. To see how the single pole-zero bound relates to the dielectric slab, we sweep the slab parameters over a wide parameter space to find the optimal advance-bandwidth product. To this end, we rewrite and simplify Eq. (2) as ${\tau }_{g,H} > -\frac{\alpha }{BW}$, which suggests that the minimum *α* can be determined through the maximum value of $-{\tau }_{g,H}BW$. We assess this value by finding, for each value of slab thickness and material parameters, the bandwidth for which the system becomes marginally stable. Without loss of generality, we first fix the material gain resonance ${\omega }_{0}$, and for each pair ($A$, $d$) we reduce $\gamma$ until a pole reaches the real axis from the lower half-plane. We then define the bandwidth as the smallest frequency for which the group advance drops below its value at $\omega \to 0$. Figure 5 shows the advance-bandwidth product, indicating that $\alpha$ remains in the order of unity. As the distance increases, for smaller values of gain in the material, i.e., a smaller *A*, we observe a mildly improved performance in terms of $-{\tau }_{g,H}BW$, but at the price of reduced group velocities that are only very moderately superluminal. For example the maximum value of $\alpha$ for $d=100{\lambda }_{0}$ occurs for a group velocity of $\approx\!1.01{c}_{0}$, corresponding to $-{\tau }_{g,H}BW\approx 2.5$, still in the same order of magnitude of the single pole-zero pair.

The dielectric slab is a good example to showcase the fundamental limits and trade-offs imposed by stability on broadband superluminal propagation, but active superluminal structures can be implemented with additional degrees of freedom in circuits. In Supplementary Discussion 1, we consider a waveguide with second-order active shunt components (a single pole/zero pair) to implement superluminal propagation, and derive a closed-form bound $\alpha < \sqrt{2}$ consistent with the previous discussion. Even when considering multiple coupled resonances tailored to dispersion engineer the response, easier to do in a circuit layout than in a dielectric structure, we find that similar bounds continue to apply (we explain more fully the involved trade-off with complexity, stability, and sensitivity of these systems in Supplementary Discussion 2). It may be possible with complex dispersion engineering to improve this bound by relying on multiple tailored resonances, but only at the price of increased complexity and footprint, and with modest overall improvements. In addition, these solutions would require a pole of the system to approach the real axis, implying the requirement of very large gain and Q-factors, as well as inherent sensitivity to small deviations from the optimal parameter designs. Practical restrictions on these quantities pose additional limits on the achievable $-{\tau }_{g,H}BW$ in realistic structures, as discussed in more depth in Sup Supplementary Discussion 3. In short, $\alpha \, < \, 1.5$ per pole/zero pair is a quantitative bound directly stemming from stability considerations that generally applies to a wide class of systems, from optics to radio-frequencies, from materials to circuit implementations, fundamentally limiting the realization of superluminal propagation over large frequency ranges and long distances. We performed an extensive review of the existing literature on active superluminal structures with reported group velocities/advances, distances, and bandwidths, finding that no experimental structure has surpassed $\alpha =1.5$ to the best of our knowledge. These structures have been implemented in a wide variety of settings, from Hz-level circuits to periodically loaded waveguides to optically-pumped and Brillouin-driven optical waveguides, yet all fall within the same range of behavior^8,10,26–29^, validating our findings. This feature speaks to the generality of the filter-theory approach derived here, but also to the difficulty of engineering a more complex dispersion response to beat the derived bound. We note that these works may not have been explicitly engineered for maximum advance-bandwidth performance, but in practice realistic systems are expected to obey this limit.

As an extreme scenario that in principle may enable a very large advance-bandwidth product, we can consider an ideal active material in which the relative permittivity and permeability are matched at all frequencies, ${\mu }_{r}={\varepsilon }_{r}$. As we show in Supplementary Discussion 4, in the ideal scenario a slab of this material can provide an unbounded advance-bandwidth product for normal incidence, thanks to the lack of reflections at its two interfaces for all frequencies. However, this comes at the price of extreme sensitivity to any mismatch from the ideal matching condition or on the incidence angle of excitation. A realistic slab with small deviations from the ideal matching condition, and/or a realistic excitation involving a finite angular range of incident radiation, would still incur self-oscillations and instabilities once we consider any minimal mismatch in the material parameters, and the inherent presence of noise triggering instabilities for eigenmodes with nonzero transverse momentum.

### Experimental verification

**Fig. 6:**
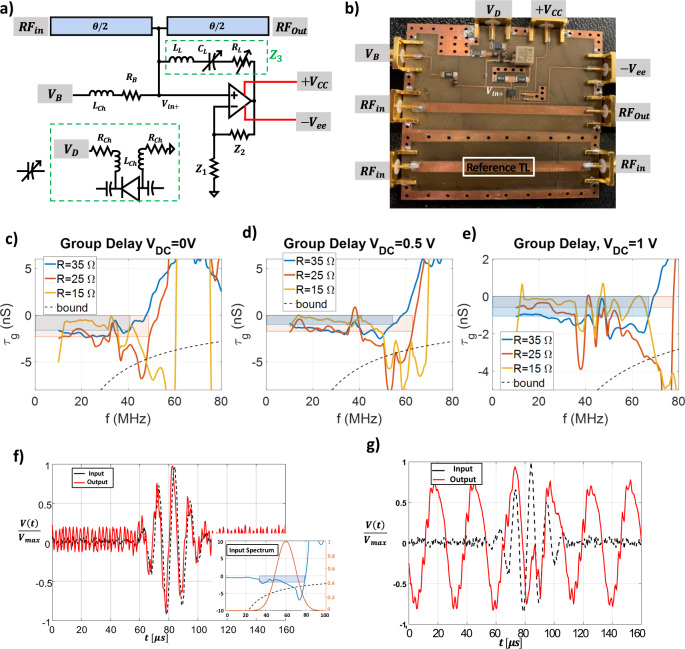
*Experimental demonstration of the proposed concept*. **a** Schematic of the fabricated circuit, a TL with electrical length $\theta$ is loaded with a shunt negative impedance implemented by a negative impedance inverter. The variable capacitance in ${Z}_{3}$ is implemented through the reversely connected varactor shown in the bottom left green box. ${V}_{b}$ is the external bias controlling the Op-Amp. **b** Fabricated PCB with FR-4 substrate. **c**–**e** Measured group delays (in ns) for different circuit parameters. **f** Time-domain measurements for a set of parameters that correspond to the spectrum shown in the bottom right inset (its group delay is shown by the dark blue line). The input Gaussian signal (black line) has a frequency spectrum (orange line) lying almost fully in the shaded blue area in the inset); the transmitted signal (red line) shows a time advance. The observed small oscillations in the output before the pulse (for $t \, < \, 60\mu s$) are due to the ring-down time from a previous pulse (repetition rate $120\,\mu s$). **g** Time-domain measurements in the unstable regime (adjusting the system parameters to the unstable regime), showing self-sustained oscillations.

In order to validate our theory, we implemented the tunable circuit shown schematically in Fig. 6a, consisting of a transmission line (TL) of electrical length $\theta$ loaded with an operational amplifier connected in shunt through the positive input terminal (${V}_{in,+}$). A detailed theoretical study of this circuit configuration is discussed in Methods and Supplementary Discussion 1. In our scenario, ${Z}_{3}$ (highlighted by the green box) consists of a series combination of an inductor (${L}_{L}$), a tunable capacitor (${C}_{L}$) and a tunable resistor (${R}_{L}$). In first approximation, this circuit implements a negative capacitor, where the variable ${C}_{L}$ controls the low-frequency group advance, and the variable ${R}_{L}$ controls the stability and bandwidth. The circuit was fabricated over a printed circuit board (PCB) using discrete components, as shown in Fig. 6(b). In addition to the main circuit, an identical unloaded TL was also fabricated on the same board as a reference, used to compare the pulse propagation to the free-space group delay ${\tau }_{0}$, coincident with the phase delay for the given TL length,${\tau }_{0}=0.4\,ns$. We use this value as a normalization constant for the group delay measurements. The first step of our experimental validation is to examine the variation in group delay as we vary the circuit parameters. Since we utilize $50\,\varOmega$ ports, the critical resistance value ${R}_{L}$ that brings the circuit to the instability threshold is ~$25\,\varOmega$ in this circuit configuration. We can independently tune the varactor diode ${C}_{L}$ (by applying different ${V}_{D}$) and ${R}_{L}$ (mechanically) to probe different regimes. We plot in Fig. 6(c–e) the measured group delay spectra after subtracting the free space delay ${\tau }_{0}$. When ${R}_{L}\approx 35\,\varOmega$ (blue lines), the circuit is well within the stable regime and it supports a moderately broadband superluminal response. For ${R}_{L}\approx 25\,\varOmega$, the system is close to the instability threshold, and for all three levels of capacitor bias this scenario supports a wider bandwidth for superluminal propagation. When ${R}_{L}\approx 15\,\varOmega$, the system has entered the unstable regime, evidenced by continuous oscillations in the time domain measurements shown in Fig. 6(g). In this regime the derived group delay is meaningless, since the system is not transmitting the input pulse but self-oscillating. In Fig. 6(c–e), we also show with shaded rectangles the corresponding group-advance bandwidth product. Due to nonidealities we do not observe the ideal response of an inverted RLC impedance, and we find additional ripples in the group-delay spectrum. By ignoring these ripples in the bandwidth calculation, taking the most generous definition of bandwidth for the advance time, we still find that the product lies well within our derived bound for any stable parameter combination we tested.

As an additional verification, we also performed time-domain measurements. First, we sent a time-domain modulated Gaussian pulse centered around $60MHz$, at which we can expect the largest negative group delay within the bandwidth of operation. Then, we adjust both ${C}_{L}$ (varying ${V}_{D}$) and ${R}_{L}$ (changing the number of turns), until we observe significant phase-advance of the transmitted signal. Figure 6(f) shows the time-domain measured signal, clearly highlighting superluminal propagation (signal in solid red). The corresponding frequency spectrum is shown in the bottom right inset together with the excitation spectrum. The measured data show a phase advance of ~1.5 ns, nicely matching the theoretical value that can be expected from the shaded blue box in the bottom right inset. Decreasing ${R}_{L}$ results in instabilities, which settle to oscillations due to nonlinearities such as gain saturation, as measured and plotted in Fig. 6(g). Slight deviations from the theoretical response are due to the Op-Amp roll-off, which results in non-ideal impedance inversion.

### Implications for active cloaking

**Fig. 7:**
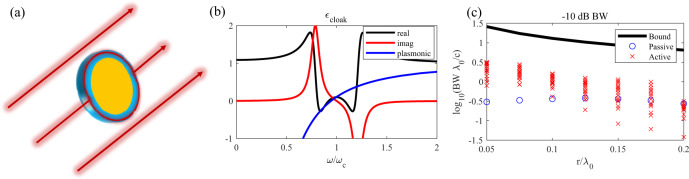
*Application of our advance-bandwidth bound to a cloaked object*. **a** A broadband superluminal cloak routes the impinging signals around an object imparting the same time delay as if the waves propagated in free space, reconstructing the incident wavefront. These waves must travel with a speed ${c}_{0}\pi /2$ in the cloak. **b** The material with a gain and loss peak can have the same permittivity at ${\omega }_{c}$, but with a flat dispersion and therefore wider cloaking bandwidth. **c** Maximum, numerically found, fractional bandwidth as a function of electrical size of the object, compared to our stability bound (black line), for a doubly resonant metamaterial cloak.

We consider now the implication of our bounds for the scenario of broadband cloaking recently discussed in Ref. ^17^. The authors suggested that a superluminal broadband cloak made of active materials may be able to suppress the scattering of an object over a bandwidth much larger than the cloaking bounds derived for passive media^14^. They argued that an active material wrapped around the object of interest may be able to exploit its superluminal speed to route a broadband pulse with the same true-time delay as if traveling in free space, as sketched in Fig. 7(a). Following the argument made in Ref. ^30^, the velocity in the cloaking region needs to be at least ${c}_{0}\pi /2$ to enable the fastest signal to traverse half of the circumference rather than the diameter of the sphere enclosing the object (we assume here that the energy is traveling as close to the perimeter as possible, otherwise even faster speeds would be required). The total travel distance is $d=\pi r$, where $r$ is the radius, and therefore ${\tau }_{g,H}=\frac{r}{{c}_{0}}\pi \left(1-\frac{2}{\pi }\right)$. Applying our inequality between group delay and bandwidth [${\tau }_{g,H}\cdot BW=\left(\frac{d}{{c}_{0}}-\frac{d}{{v}_{g}}\right)BW \, < \, 1.5$], we find that $r\cdot BW < 1.3{c}_{0}$. Interestingly, this bound is of the same form as the limits for passive scatters derived in Ref. ^14^, even though, different from the general passivity bounds in Ref. ^14^, our derivation here stems from a ray optics picture specifically tailored for impenetrable objects^31,32^.

To test the applicability of our bound, we consider the case of a subwavelength 3D perfectly conducting (PEC) sphere, previously examined in Ref. ^33^. The scattering from a subwavelength structure cannot be described with ray optics, but, as we show in the following, it is found to exhibit the same tradeoff between size and bandwidth, owing to stability considerations. The dominant scattering term in such a particle is the electric dipole mode, which can be effectively suppressed under the condition ${\varepsilon }_{cloak}\approx \frac{{x}^{3}-1}{{x}^{3}+2}$, where $x$ is the ratio of outer radius of the cloaking layer to the sphere radius. Notably, this condition on ${\varepsilon }_{cloak}$ is independent of the particular frequency, and requires^34^ that its value is <1. That is, to make a broadband cloak, we need a superluminal permittivity over a wide frequency range, exactly the kind of behavior we have discussed thus far. Such behavior may be enacted with a causal dispersion by operating between a loss and a gain resonance. Although there are a number of free parameters to explore, we will study the behavior under the dispersion profile

5 $${\varepsilon }_{cloak}=1+A{\omega }_{c}\left(\frac{-{\omega }_{1}}{{{\omega }_{1}}^{2}-{\omega }^{2}-i\gamma \omega }+\frac{{\omega }_{2}}{{{\omega }_{2}}^{2}-{\omega }^{2}-i\gamma \omega }\right)$$

where ${\omega }_{1}={\omega }_{c}+\varDelta \omega /2$ corresponds to the higher-frequency gain resonance and ${\omega }_{2}={\omega }_{c}-\varDelta \omega /2$ to the lossy resonance. This particular dispersion provides a flat ($\frac{\partial \varepsilon }{\partial \omega }=0$) and essentially lossless (${{\mbox{Im}}}[\varepsilon ]=0$) dispersion at ${\omega }_{c}$, desirable for broadband cloaking. An example of such a dispersion profile is shown in Fig. 7(b), compared to a lossless Drude-like material ($\varepsilon =1-{{\omega }_{p}}^{2}/{\omega }^{2}$). For any given $\varDelta \omega$ and $\gamma$, the oscillator strength $A$ can be chosen to meet the cloaking condition at ${\omega }_{c}$. However, as $\varDelta \omega$ increases to enhance the bandwidth, $A$ must increase as well, making the system more susceptible to instabilities. As clear in Fig. 7(b), the dispersion of the active material offers the potential for a flatter dispersion and broader cloaking than any passive medium, considering that the Drude dispersion is the least dispersive material without gain.

To explore the tradeoff between scattering suppression, bandwidth and size, we fix ${\omega }_{c}=2\pi \times 200THz$ and $x=1.1$, and for a given PEC sphere with radius *r* we increase $\varDelta \omega$ and the corresponding $A$ until the system develops an instability. We parametrically sweep $\gamma$ from $0.001{\omega }_{c}$ to $0.5{\omega }_{c}$ for each electrical size and plot the corresponding bandwidth of scattering reduction in Fig. 7(c), together with the same calculation in the case of a Drude cloak. The bandwidth is defined as the frequency range over which a 10 dB reduction of scattering cross section is observed in comparison to the bare PEC sphere, both calculated with Mie theory^35^. The active cloak can indeed provide a larger bandwidth for all considered geometries compared to a passive cloak, but both stay well below the stability bound discussed here.

**Fig. 8:**
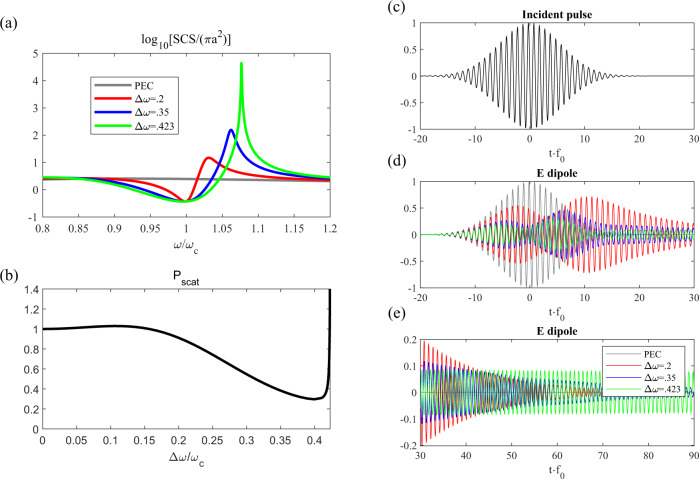
*Performance of active band-pass cloak for a PEC sphere with*$r=0.2{\lambda }_{0}$. **a** Scattering cross section for the PEC sphere and active cloaks with different resonance features. The wider resonance separation generally increases both bandwidth and out-of-band scattering. **b** Total scattered power (normalized to the bare sphere) as a function of the resonance separation. **c** Incident plane-wave pulse at the center of the sphere and **d** induced electric dipole scattering for different cloak widths (normalized to the maximum instantaneous value of the PEC). **e** Temporal evolution of the dipolar scattering after the pulse excitation, showing clear ringing as the system approaches instability ($\varDelta \omega \approx 0.42{\omega }_{c}$).

Increasing $\varDelta \omega$ enhances the cloaking bandwidth, as shown in Fig. 8(a), but the out of band scattering near the gain resonance increases, until the stability threshold is crossed. The overall behavior clearly falls within our bound [black line in Fig. 7(c)], with the same $BW\propto 1/r$trend. We note that changing the cloaking reduction level changes the bandwidth that the structure is able to attain, but the same fundamental tradeoff between size and bandwidth is expected.

We can also explore the effects of the cloak on the scattering of a pulse of finite temporal duration. In Fig. 8(b), we show the effect of increasing $\varDelta \omega$ on the total scattered power for a Gaussian pulse of fixed temporal and spectral extent, whose time-domain field is plotted in Fig. 8(c), chosen to fit within the cloaking bandwidth available before instabilities set in. Compared to the PEC sphere, the cloaked object scatters less for certain bandwidths, but then the scattering spikes up as the system approaches the instability threshold. Figure 8(d, e) show the temporal evolution of the dominant (dipolar) scattering component $p(\omega )=\frac{-6\pi i}{{{k}_{0}}^{3}}{E}_{in}(\omega ){C}_{1,TM}(\omega )$^35^ for various values of $\varDelta \omega$. Overall, the active cloaks successfully suppress the scattering over the main duration of the incident pulse. However, due to the presence of gain that brings the pole closer to the axis, there is a significant ringing after the incident pulse has subsided. When the pole comes to the real axis, this ringing is sustained indefinitely, corresponding to infinite scattered power.

This example demonstrates how the behavior we derived in a 1D wave-optics picture is more generally observed also in 3D cloaking problems. Larger cloaks based on transformation optics generally require more complex inhomogeneous and anisotropic permittivity and permeability profiles, but our arguments are expected to still apply. Refs. ^33,36^ have also explored stability considerations in active scattering systems, pointing out related limitations. Overall, the theoretical proposal in Ref. ^17^ for broadband cloaking of large objects based on superluminal propagation does not consider the inherent instabilities that these objects necessarily run into as their size grows, implying that the cloaked objects would become not only more visible, but actually likely lead to lasing and bright self-oscillations rather than cloaking.

## Discussion

In this paper, we derived a quantitative relationship between superluminal velocity, bandwidth, and maximum propagation length in active materials, dictated by stability considerations, applicable to a wide range of implementations, from metamaterial devices to circuit networks. While active structures may be causal, they are prone to instabilities when striving for extreme responses. Our derived bound indicates that the group advance-bandwidth product is limited to order unity in any realistic material and circuit layout supporting superluminal responses, hence it cannot be used for arbitrarily broadband cloaking of large objects or other extreme functionalities. Signal distortion, expected when operating close to our derived bound, poses even more stringent limitations on the bandwidth of operation and group advance in these systems.

## Methods

### Experimental implementation

The negative capacitance in our experiment is implemented by configuring the operational amplifier (Op-Amp) as a negative impedance converter. The input impedance seen at the positive Op-Amp terminal is $-{Z}_{3}\frac{{Z}_{1}}{{Z}_{2}}$, i.e., by selecting ${Z}_{1}={Z}_{2}=500\,\varOmega$ we obtain the input impedance $-{Z}_{3}$. The load ${Z}_{3}$ is a series RLC, with tunable $R{}_{L}$ and ${C}_{L}$, and a fixed ${L}_{L}\approx 51\,nH$.6(a). The tunable capacitor is implemented with a varactor diode (SMV12555, Skyworks) reversely biased, as shown in the green box in Fig. 6(a) through the tuning voltage ${V}_{D}$. The capacitance is tuned by${V}_{DC}$ through the nonlinear relation $C={C}_{0}{(1+\frac{{V}_{DC}}{{V}_{j}})}^{-M}$ where the parameters ${C}_{0}$,${V}_{j}$, and $M$ are $80\,pF$, $135\,V$, and $100$, respectively (SMV12555, Skyworks). In order to introduce full on-board tunability and adjustment for the circuit (and to properly DC-bias the Op-Amp), the bias voltage ${V}_{B}$ is introduced to DC-bias the positive input terminal through the biasing resistor ${R}_{B}=10k\varOmega$ and the RF choke inductor ${L}_{ch}=4.7\mu H$. The tunable resistor is implemented using an SMD trimmer resistor (PVG5H201C03R00, Bourns) through on-board mechanical tuning by a screwdriver, with a full range of $200\,\varOmega$. The corresponding S-parameters were extracted using a vector network analyzer (VNA).

## Supplementary information

Supplementary Materials

## Data Availability

All data generated or analyzed during this study are available upon contacting the corresponding author.

## Code availability

Codes used for this study are available upon request by contacting the corresponding author.
