# Supplementary material for: Stability bounds on superluminal propagation in active structures
Source: Nat Commun. 2022 Mar 2;13:1115. doi: 10.1038/s41467-022-28713-x (PMC8891327; doi:10.1038/s41467-022-28713-x)
Supplement: Supplementary file 1 — Supplementary Materials [file 41467_2022_28713_MOESM1_ESM.pdf]

Supplemental Material for "Stability-induced bounds on superluminal propagation in active structures"

Robert Duggan,[1,2] Hady Moussa,[2,3] Younes Radi,[2] Dimitrios L. Sounas,[4] and Andrea Alù[1,2,3,5,*]

[1]Department of Electrical and Computer Engineering, The University of Texas at Austin, Austin, Texas 78712, USA

[2]Photonics Initiative, CUNY Advanced Science Research Center, New York, NY 10031, USA

[3]Department of Electrical Engineering, City College of the City University of New York, New York, New York 10031, USA

[4]Department of Electrical and Computer Engineering, Wayne State University, Detroit, MI 48201, USA

[5]Physics Program, Graduate Center, City University of New York, New York, New York 10026, USA

[*]aalu@gc.cuny.edu

Supplementary Discussion

1. Bound derivation in shunt negative RLC element

As a representative model, consider an air loaded lossless transmission line with propagation constant $\beta = \omega/c_0$, loaded with a single shunt series RLC element connected through a negative impedance converter, which can be achieved with a cross-coupled pair of transistors, for example[1,2], as sketched in Supplementary Figure 1. In the main text, we implement the negative impedance with a properly configured Op-Amp topology. Such a system effectively implements a negative capacitor, which may be used to realize superluminal propagation. Its relative simplicity allows us to evaluate in closed-form the response of the system, and its limitations in terms of superluminal operation. For simplicity, we neglect the dispersion of the lumped elements, so we

have a shunt admittance $Y_{shunt}(\omega) = -Y_{RLC}(\omega)$. We assume an ideal NIC, but by including the inductor in the shunt element (which is sometimes ignored), the admittance stays causal (in time domain, no current flows before a voltage is present). We also assume the terminations to match the waveguide impedance Z_0. Although different from the standard circuit convention, we will use the $e^{-i\omega t}$ convention to be consistent with the main text. The transfer function through a length d is given by

$$T = e^{id\omega/c_o} \frac{1 + j\omega RC - LC\omega^2}{1 + j\omega(R - Z_0/2)C - LC\omega^2}. \tag{S.1}$$

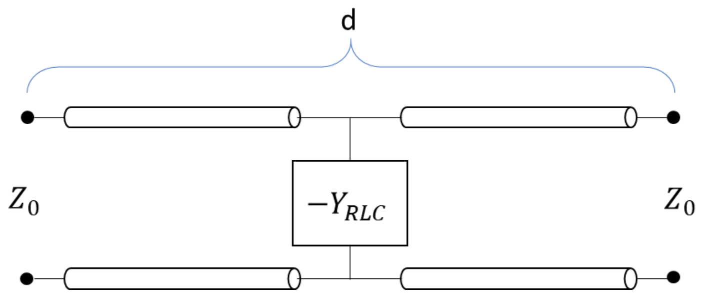

Supplementary Figure 1- *Circuit model of the considered setup*. A negative series RLC connected through a negative impedance converter forms a shunt admittance in a waveguide of total length d, characteristic impedance Z_0 and velocity c_0. The port impedances are matched to the waveguide.

The poles of the system are given by

$$\omega_{pole} = \frac{-i(R - Z_0/2)}{2L} \pm \sqrt{\frac{1}{LC} - \frac{(R - Z_0/2)^2}{4L^2}}, \tag{S.2}$$

and the zeros by

$$\omega_{zero} = \frac{-iR}{2L} \pm \sqrt{\frac{1}{LC} - \frac{R^2}{4L^2}}, \tag{S.3}$$

where all parameters are positive. Stability is achieved when the imaginary part of all poles is ≤ 0, requiring that $R_{eff} = R - Z_0/2 > 0$. This condition can be simply understood as the requirement that the total resistance of the parallel combination of the ports and active element must stay positive. For this reason, we define $R_0 = Z_0/2$ as the critical resistance for stability. If an additional series resistance R_{wg} is added to the circuit, possibly modeling radiation loss in the case of leakage into free-space due to the superluminal velocity (as in a leaky-wave antenna or in the case of Cherenkov radiation), we can define a modified $R_{eff} = R - Z_0 \frac{R_{wg} + Z_0}{R_{wg} + 2Z_0}$, but otherwise the following analysis is unchanged. It is important to remember that any system must remain stable to actually operate as a waveguide, or else the behavior will more resemble an oscillator once nonlinearities and saturation comes into play.

We calculate the phase of T and derive an effective phase velocity through the accumulated phase shift as the signal is transmitted through the circuit. The group delay is given by the frequency derivative of the phase of (S.1) and the effective velocity is the distance divided by this quantity. The group velocity is explicitly

$$v_g = \frac{1}{\frac{1}{c_0} - \frac{C Z_0 \left(1 + C L\omega^2\right)\left(2 + C\omega^2\left(Cr\left(-2R + Z_0\right) + 2L\left(-2 + CL\omega^2\right)\right)\right)}{d\left(1 + C\omega^2\left(C R^2 + L\left(-2 + C L\omega^2\right)\right)\right)\left(4 + C\omega^2\left(C\left(-2R + Z_0\right)^2 + 4L\left(-2 + CL\omega^2\right)\right)\right)}}. \tag{S.4}$$

The phase and group velocities are nearly flat at low frequencies, with low-frequency value

$$v_{p,g}\left(\omega=0\right)=\frac{1}{\frac{1}{c_0}-\frac{CZ_0}{2d}}. \tag{S.5}$$

Interestingly, this expression matches the form of Eq. (2) in the main text, so by inspection we obtain $BW \le 2\alpha/CZ_0$, which relates the maximum allowed bandwidth over which the velocity can be superluminal to the material properties. An upper bound on this low-dispersion superluminal bandwidth is the resonance frequency of the pole, at which the system becomes most dispersive and we therefore expect a large, positive delay. Equation (S.4) can be simplified if we combine it with the stability limit $R=R_0$. By taking the product of $\omega_p=\frac{1}{\sqrt{LC}}$ and the minimum group velocity over the bandwidth, which occurs either at $\omega=0$ ($\tau_g=C\,Z_0/2$) or $\omega=\omega_p$ ($\tau_g=4L/Z_0$), that is $v_{g,\min}=\frac{1}{\sqrt{LC}}Min\left[C\,Z_0/2\,,4L/Z_0\right]$, we obtain the maximum product for an inductance that results in $BW=2\sqrt{2}/C\,Z_0$. This clearly provides $\alpha = \sqrt{2}$ by inspection with (S.5), in perfect agreement with the generic result found with filter theory under the limit of unitary transmission at $\omega=0$.

In Supplementary Figure 2, we study the effect of the circuit parameters R , L and C on the response of the system. In (a), L is chosen to maximize the bandwidth for given C , and then R is brought closer to the critical stability value. It is clear that to bring the superluminal bandwidth near the limits stated by the bound, the system must be brought closer to instabilities. These instabilities may be triggered if the actual system parameters deviate from the intended design, and there will also be enhanced noise from these elements that provide significant amplification to the system. Supplementary Figure 2(b) shows the role that L plays in this

response: inductor values that are too large result in a reduced bandwidth, but values that are too low result in a depressed velocity within the bandwidth. In (c), we keep R constant and vary C, while also varying L to keep the bandwidth maximized. We see that there is a tradeoff between the velocity and bandwidth that tracks nicely with the bound we have developed in the main text.

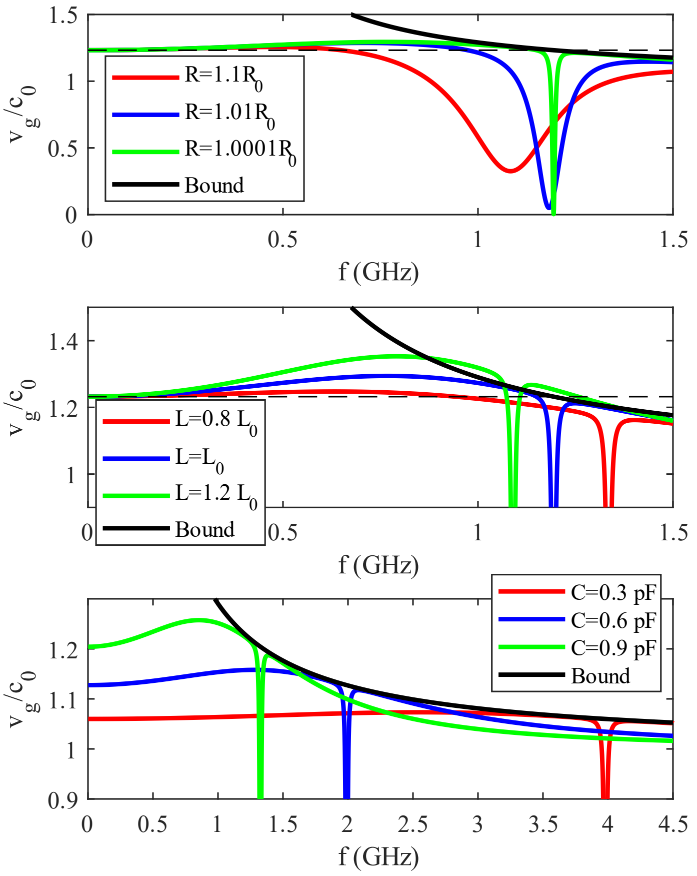

Supplementary Figure 2- *Effect of varying circuit parameters*. (a) Normalized group velocity versus frequency as the value of R is changed. The inductance is varied to maximize bandwidth for given R. $C=1pF$, $d=30cm$. The bandwidth suffers significantly as R is varied from the optimal value. The dashed black line indicates the group velocity as $\omega=0$. (b) Group velocity dispersion for variable levels of inductance for $C=1pF$, $d=30cm$ and $R=1.0001R_0$. (c)

Dispersion curves for various capacitor values, and $R = 1.0001 R_0$. The inductance is varied to maximize bandwidth for given C.

2. Limits on bandpass RLC setup

It is also possible to leverage two or more resonances to observe band-pass dispersionless superluminality, with superluminal behavior arising from more complex gain responses[3]. We can easily extend our model to have a second shunt element in parallel with the first one, assuming negligible electrical length between them. The characteristic equation for the transmission coefficient poles is 4th order in this case, and the minimal resistance values to achieve stability vary based on other parameters. Generally, the minimum resistance is $Z_0/2$ in the limit of large difference in the resonance frequencies, but it becomes Z_0 for resonance frequencies close together. Although the closed form expression for the poles is quite complicated, we can arrive at a simplified form under assumptions consistent to realistic working conditions. We note at first that the zeros of S_{21} are the zeros of each shunt RLC, as in (S.3). If we assume the same value of L in each resonator, and take R to the $Z_0/2$ stability limit in both, which we have shown is needed to maximize the bandwidth in the low-pass scenario, then the zeros will have the same imaginary parts $\omega_{i,z}$ and will differ in the oscillating terms, given by $\omega_{z,r,0} \pm \Delta\omega_{z,r}$. Under these assumptions, the poles of the transfer function can be explicitly written as $\omega_p = \pm \frac{i}{2}\sqrt{-\Delta\omega_{z,r}{}^2 + 4\left(\omega_{i,z}{}^2 - \omega_{z,0}{}^2 \pm \sqrt{-4\omega_{i,z}{}^2\omega_{z,0}{}^2 + \Delta\omega_{z,r}{}^2\left(-\omega_{i,z}{}^2 + \omega_{z,0}{}^2\right)}\right)}$.

For $\Delta\omega_{z,r} < 2\omega_{i,z}$, the poles have imaginary parts, and so will lie on either side of the imaginary s-axis and be unstable; otherwise, all of the poles lie on the imaginary axis and the

system is marginally stable, becoming absolutely stable once $R > Z_0/2$. We assume to work in the marginally stable regime and, similar to the low-pass case, we define the bandwidth as the frequency difference between the two positive-frequency poles. We can also use geometric arguments analogous to the previous section to calculate the relative group advance at the center of the frequency range, based on the positions of the zeros. Combining them, we find the figure of merit

$$-\tau_{g,H} \cdot BW = \frac{4\omega_{i,z} \operatorname{Re}\left[\begin{array}{l} \sqrt{\Delta\omega_{z,r}{}^2 + 4\left(-\omega_{i,z}{}^2 + \omega_{z,r,0}{}^2 + \sqrt{-4\omega_{i,z}{}^2\omega_{z,0}{}^2 + \Delta\omega_{z,r}{}^2\left(-\omega_{i,z}{}^2 + \omega_{z,r,0}{}^2\right)}\right)} - \\ \sqrt{\Delta\omega_{z,r}{}^2 - 4\left(\omega_{i,z}{}^2 - \omega_{z,r,0}{}^2 + \sqrt{-4\omega_{i,z}{}^2\omega_{z,0}{}^2 + \Delta\omega_{z,r}{}^2\left(-\omega_{i,z}{}^2 + \omega_{z,r,0}{}^2\right)}\right)} \end{array} \right]}{\Delta\omega_{z,r}{}^2 + 4\omega_{i,z}{}^2}.$$

(S.6)

In the limit of $\omega_{z,r,0} \gg \omega_{i,z}, \Delta\omega_{z,r}$, the series expansion shows that this function is maximum at $\Delta\omega_{z,r} = 2\sqrt{3}\omega_{i,z}$, and provides a value of $\sqrt{2}$, exactly the same as the low-pass case. Notice in this case that the poles end up being narrower than the zeros, and we end up with the same total frequency bandwidth for a given delay, even with a more complex circuit topology. This appears to indicate that our derived bounds provide a good sense of the limits in practical situations, and more complex dispersion engineering may not be feasible in practical scenarios. In Supplementary Figure 3, we show an example of the band-pass superluminal dispersion, along with the placement of poles and zeros in these optimized setups. It is clear that the dispersive "dips" in velocity correspond to the position of the poles, which are close to the real frequency axis, whereas the broad superluminal curves peak around the position of the zeros, which lie further off the axis.

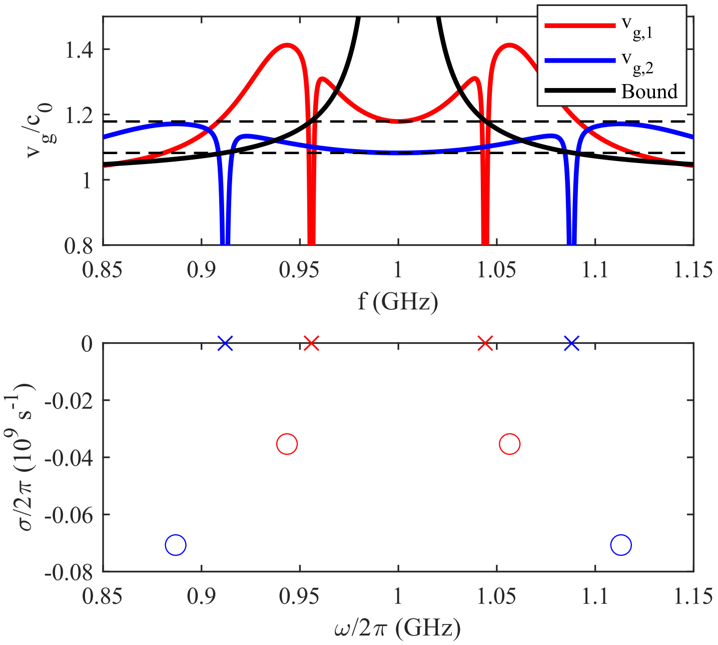

Supplementary Figure 3 - *Band-pass superluminal setup based on two resonators.* (a) Group velocity dispersion with center frequency $f_0 = 1GHz$, $d = 2m$, $R_1 = R_2 = 1.001R_0$. Due to the dispersion near resonance, the bandwidth is exactly the same as in the low-pass scenario analyzed in Supplementary Figure 1, and it holds for any value of center frequency and velocity. The dashed black lines indicate the group velocities at the center frequency. Red corresponds to a design for $80\,MHz$ bandwidth and blue corresponds to $160\,MHz$. (b) Poles (x) and zeros (circles) in the complex frequency plane for the two setups.

3. Effects of limited gain and Q factor

So far, we have explored the bounds on group velocity defined as the velocity of a pulse peak under the assumption of narrowband excitation, corresponding to $\partial\omega / \partial k$. When the pulse is sufficiently broadband compared to the dispersion of the structure, the correspondence between these two quantities may break down, due to significant distortions as the pulse progresses in the medium. As discussed in[4,5], pulse distortion adds practical limits that are in general stricter than

the incurrence of instabilities on which we focus in the main paper. Kitano et. al[6] found an advance-bandwidth product of roughly unity. To approach this bound and the similar bounds derived in our work, a pole must be brought very close to the real frequency axis, acting more like an oscillator rather than a transmission line. In addition, these conditions also require large gain, which is a practical challenge and may result in nonlinearities becoming important, such as gain saturation. The obvious question is how any restrictions on the amplitude of T limit the superluminal behavior. We consider two approaches, with results provided in Supplementary Figure 4. In the first, we set a global amplitude maximum for T, and then optimize the delay-bandwidth product similar to the previous sections, imposing unitary transmission at $\omega = 0$ to be consistent with the RLC scenario of Supplementary Figure 1. As expected, the bound is approached asymptotically as the global maximum grows, with about a factor of 10 needed to achieve half of the bound. Alternatively, we optimize $\tau_{g,H} \cdot BW$ as a function of $\omega_{r,pole} / \omega_{i,pole}$, proportional to the system Q -factor and normalized ring-down time. Again, the bandwidth asymptotically approaches the limit as Q increases. The product reaches only half of its maximum value for $Q = 10$, which is certainly expected to produce significant distortion in a signal with a spectral component at resonance. Alternatively, a latency period of 10 oscillation cycles is needed to prevent leakage into the next pulse.

It should also be pointed out that there is no restriction stemming from stability or causality to having multiple zeros in the superluminal bandwidth, in which case $-\tau_{g,H} \cdot BW$ can be increased with each pole-zero pair. By placing n sets of identical poles and zeros, the product can grow as $1.5n$. In theory, then, there is no absolute bound to the group delay-bandwidth product based on the combined contribution of a large number of pole-zero pairs. This will, however, require

complex waveguide implementations and dispersion engineering. Any particular circuit topology may present additional practical restrictions not captured in our general filter theory, as evident in the case of the two RLC elements discussed previously. In other words, adding additional elements to the system is no guarantee of being able to actually increase the overall advance-bandwidth product, as there may not be enough independent degrees of freedom to arbitrarily tailor the positions of each pair. In addition, distortion effects will be enhanced with additional pole-zero pairs: in the limit of a pair made doubly-degenerate, the group advance would double, but the transmission maxima will be squared.

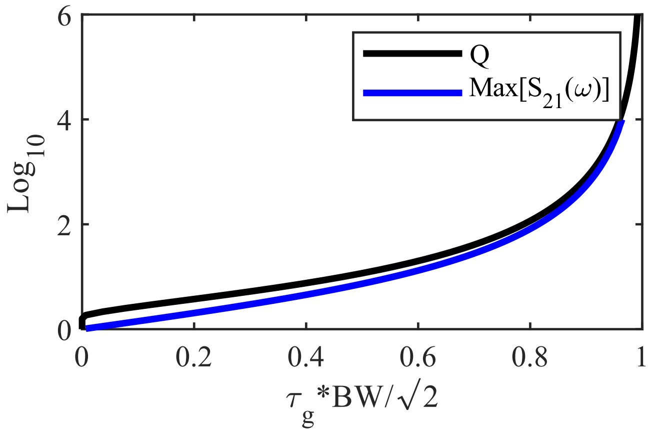

Supplementary Figure 4- *Effect of finite Q or gain.* Numerically calculated relationship between the advance-bandwidth product and the maximum allowed transmission gain, or the minimum Q-factor needed, for the low-pass circuit of Supplementary Figure 1.

4. Case of matched $\mu_r = \varepsilon_r$

The instabilities that arise in active structures and fundamentally limit their response are due to feedback from mismatch at the interfaces of the active structures and the passive ports. Under certain restrictions, such as of a purely dielectric response, these mismatches are unavoidable. In

theory, though, one can envision the ideal case in which the magnetic response follows precisely the same frequency response as the electric permittivity. In this case, the impedance of the slab is equal to the impedance of free space, there will be no reflections at the interfaces, and therefore no feedback to cause instabilities. Can this behavior fit within the filter theory approach outlined in the main text? The answer is yes: this ideal scenario includes a very peculiar singularity for which there are poles and zeros of infinite order that simultaneously lie at the same point in the complex ω plane. In one example of such a matched system where we use the permittivity of the slab in the main text and the same relative permeability, the transmission coefficient may be written as

$$T = e^{\frac{i\omega d}{c_0}} e^{\frac{i\omega d}{c_0}\frac{A\omega_0^2}{\omega_0^2-\omega^2-i\gamma\omega}} \tag{S.7}$$

with $H(\omega) = e^{\frac{i\omega d}{c_0}\frac{A\omega_0^2}{\omega_0^2-\omega^2-i\gamma\omega}}$. Different from the polynomial case, here poles and zeros are obtained when the exponent goes to $+\infty$ or $-\infty$ respectively, and the poles and zeros are of infinite order, due to the ideal matching of the material at all frequencies. In this case, we have an unsigned infinity at the complex frequencies $\omega = -\frac{i\gamma}{2} \pm \sqrt{\omega_0^2 - \gamma^2/4}$, although the complex nature of the infinity (positive or negative real/imaginary parts) is determined by the direction from which the point is approached. For example, in the case of gain ($A < 0$), the $+\infty$ "pole-like" response is found when this point is approached from the side of the real axis, and the $-\infty$ "zero-like" response is found when this point is approached from the away from the real axis [see Supplementary Figure 5(b)]. Note that the poles are pinned to this position in the complex plane, and so this slab never becomes unstable as the length is increased (this is limited to normal incidence). The infinite order of these poles makes up for the fact that they are infinitesimally close together, so that the net

effect is superluminal velocities for frequencies away from resonance when the poles are “too close” to the axis and have a smaller angular derivative than the zeros, but subluminal velocities when the frequencies are near the resonance. In essence, the zeros “feel” further from the axis and provide a broadband, superluminal response, whereas the poles are closer to the axis and provide stronger subluminal response near resonance. This is exactly the behavior we see when calculating the group velocity, shown in Supplementary Figure 5(d). Practically, achieving such a response with perfectly matched magnetic and electric properties at all frequencies is totally unrealistic, and any small deviation would imply the emergence of instabilities. The perfect matching also no longer holds when the incident angle is not at normal incidence, and so the feedback necessary for oscillations becomes present again. For example, with the same matched slab, at 5° incident angle, the transmission coefficient clearly shows poles in the upper half-plane Supplementary Figure 5(f), and so the structure may oscillate with emission at those angles. Still, this example highlights that it is not strictly forbidden with extreme dispersion engineering to achieve advance-bandwidth products significantly larger than unity.

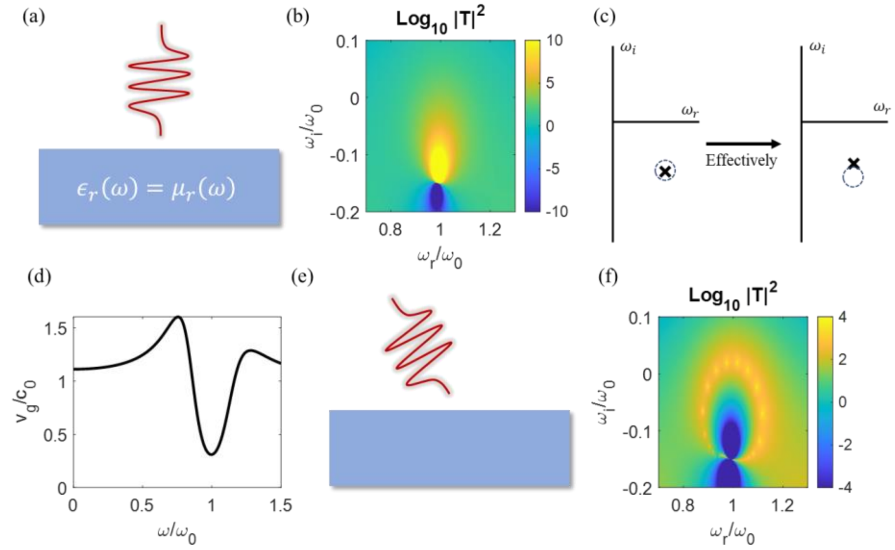

Supplementary Figure 5- *Superluminal matched slab*. (a) Schematic of the situation under consideration of normal incidence upon a slab that is matched at all frequencies, such that there are no reflections at the interfaces. (b) Log10 transmitted power of the matched slab near the singularity, with large "pole-like" responses on the real-frequency axis side of the singularity and the "zero-like" response on the opposite side. The plotted values have been clipped between [-10,10] for presentation. (b) Sketch of the effective behavior of the poles and zeros, where although they truly lie at the same frequency, in the calculation of superluminal velocities they effectively are split apart. (d) Plot of the group velocity, with superluminal behavior away from resonance and subluminal propagation near the resonance. (e) Schematic of the case of off-normal incidence, such that reflections at the interfaces are now possible. (f) The calculated transmission coefficient at 5° incidence off normal, where the diverging spot indicate the locations of the poles, some of which have moved into the upper half plane, indicating instability. Values clipped between [-4,4] for presentation purposes. Parameters used: $\gamma = 0.3\omega_0$, $A = -0.1$, $d = 4c_0/(\omega_0/2\pi)$.

Supplementary References

1. Razavi, B. The cross-coupled pair-part III [A circuit for all seasons]. *IEEE Solid-State Circuits Mag.* **7**, 10–13 (2015).

2. Kord, A., Sounas, D. L. & Alù, A. Active Microwave Cloaking Using Parity-Time-Symmetric Satellites. *Phys. Rev. Appl.* **10**, (2018).

3. Steinberg, A. M. & Chiao, R. Y. Dispersionless, highly superluminal propagation in a medium with a gain doublet. *Phys. Rev. A* **49**, 2071–2075 (1994).

4. Garrett, C. G. B. & McCumber, D. E. Propagation of a Gaussian light pulse through an anomalous dispersion medium. *Phys. Rev. A* **1**, 305–313 (1970).

5. Long, J. & Sievenpiper, D. F. The Observation of Dispersionless Superluminal Propagation in a Non-Foster Loaded Waveguide and Its Fundamental Limitations. *IEEE Trans. Microw. Theory Tech.* 1–12 (2017) doi:10.1109/TMTT.2017.2769048.

6. Kitano, M., Nakanishi, T. & Sugiyama, K. Negative group delay and superluminal propagation: an electronic circuit approach. *IEEE J. Sel. Top. Quantum Electron.* **9**, 43–51 (2003).
